# Effects of Knee Joint Angle on Intramuscular Blood Flow and Muscle Deoxygenation During Fatiguing Isometric Contractions

**DOI:** 10.1111/sms.70269

**Published:** 2026-04-02

**Authors:** Kazuma Izumi, Keisho Katayama, Yutaka Kano, Noriko Tanaka, Hiroshi Akima

**Affiliations:** ^1^ Graduate School of Education and Human Development Nagoya University Nagoya Aichi Japan; ^2^ Research Center of Health, Physical Fitness and Sports Nagoya University Nagoya Aichi Japan; ^3^ Graduate School of Medicine Nagoya University Nagoya Aichi Japan; ^4^ Department of Engineering Science, Bioscience and Technology Program University of Electro‐Communications Chofu Tokyo Japan

**Keywords:** fatiguing exercise, intramuscular blood flow, knee joint angle, muscle deoxygenation, muscle oxygen consumption, power Doppler ultrasonography

## Abstract

The purpose of this study was to investigate the effect of the knee joint angle on intramuscular blood flow and muscle deoxygenation during fatiguing intermittent isometric knee extensions. Seventeen healthy young male participants (19.6 ± 1.3 years) performed intermittent (5 s contraction, 5 s relaxation) and incremental isometric knee extensions at 30%, 40%, 50%, 60%, and 70% of maximal voluntary contraction (MVC) consecutively until task failure. Exercise was performed at flexed (90°) and extended (140°; full extension = 180°) knee joint angles. Intramuscular blood flow and muscle oxygen saturation (StO_2_) were measured using power Doppler ultrasonography and near‐infrared spectroscopy, respectively, in the right vastus lateralis at mid‐thigh. Although MVC torque did not differ significantly between knee joint angles (*p* = 0.139), endurance time was significantly longer in the extended than in the flexed position (*p* = 0.002). There were no significant differences in intramuscular blood flow (*p* = 0.105) and StO_2_ (*p* = 0.292) between knee joint angles at baseline. The Δintramuscular blood flow was significantly higher in the flexed than in the extended position from 30% to 60% MVC (*p* = 0.006), and ΔStO_2_ was significantly lower in the flexed than in the extended position above 30% MVC (*p* = 0.026). These results indicate that changes in intramuscular blood flow and muscle deoxygenation are influenced by knee joint angle at submaximal intensities during intermittent isometric knee extension exercise.

## 1 Introduction

Oxygen demand increases in active muscles, and muscle oxygen consumption (mV̇O_2_) rises during exercise [1]. As is well known, knee joint angles during knee extension affect mV̇O_2_. In fact, mV̇O_2_ in the vastus lateralis has been reported to be higher in the flexed than in the extended position during repetitive isometric knee extension exercise [2, 3, 4, 5]. For example, Kooistra et al. [2] compared mV̇O_2_ at 90°, 120°, and 150° (with 180° indicating full knee extension) during repetitive isometric knee extension. They found that mV̇O_2_ at 150° was significantly lesser than at 90° and 120°, suggesting that muscle oxygen demand is lower even at identical relative intensities and that oxygen supply may be more effective [5]. The increase in metabolic cost (i.e., mV̇O_2_) is thought to contribute to greater fatigue in the flexed than in the extended knee joint angle [2]. Consistent with this, studies have shown that time to exhaustion is shorter [6] and knee extension force during repetitive fatiguing contractions declines further [7] in the flexed than in the extended position during isometric knee extension.

Changes in muscle and capillary structure associated with the joint angle may underlie the differences in metabolic cost and muscle endurance. Compared with a flexed knee angle, muscle fascicle length is shorter and the pennation angle is larger in the extended position [8, 9]. A greater increase in the pennation angle has been associated with a larger reduction in regional blood volume during muscle contraction [10]. It is thought that when fascicle length decreases and the pennation angle increases, the force component perpendicular to the aponeuroses also increases, resulting in higher intramuscular pressure [11]. Therefore, we expected that intramuscular blood flow during muscle contraction would be more restricted—and post‐exercise hyperemia more pronounced—in the flexed than in the extended position.

Conversely, capillaries may be more stretched at longer sarcomere lengths, which decreases capillary lumen diameter and red blood cell velocity [12]. Moreover, mV̇O_2_ increases more in the flexed than in the extended position [3, 4]. Taken together, these findings led us to believe that intramuscular blood flow may be greater in the flexed than in the extended position. However, whether the knee joint angle during isometric knee extension affects changes in intramuscular blood flow and muscle deoxygenation has not been clarified, likely because of methodological limitations in assessing intramuscular blood flow during exercise.

Power Doppler ultrasonography, which allows non‐invasive assessment of tissue perfusion, is widely used in clinical settings for diagnosing and monitoring organ and musculoskeletal conditions [13, 14]. We have applied power Doppler ultrasonography to assess intramuscular blood flow [15, 16, 17]. This technique provides the integrated power of the complete Doppler spectrum and detects frequency shifts derived from red blood cell flow [18]. Thus, power Doppler ultrasonography enables non‐invasive, continuous evaluation of intramuscular blood flow.

The purpose of this study was to examine the effect of knee joint angle on intramuscular blood flow and muscle deoxygenation in the vastus lateralis during fatiguing isometric knee extension exercise. Because mV̇O_2_ has been reported to be higher in the flexed than in the extended position, [4] we hypothesized that intramuscular blood flow would be higher and muscle oxygen saturation (StO_2_) would be lower in the flexed than in the extended knee joint angle.

## 2 Methods

### 2.1 Participants

This study involved 17 healthy male participants (age, 19.6 ± 1.3 years; height, 171.1 ± 5.9 cm; weight, 60.2 ± 5.7 kg). This study was approved by the Ethics Committee of the Research Center of Health, Physical Fitness & Sports at Nagoya University (Approval No. 21–05) and conformed to the standards set by the Declaration of Helsinki. Before the experiments, the purpose, risks, and benefits of the study were explained, and informed consent was obtained from all participants.

### 2.2 Experimental Procedures

All participants visited the laboratory on three separate days with an interval of at least two days. On the first visit, they practiced maximal voluntary contraction (MVC) and familiarized themselves with the intermittent isometric knee extension tasks. On the second and third experimental days, they performed MVC measurements and fatiguing isometric knee extension tasks with the right knee joint angle set at 90° (flexed position) or 140° (extended position; 180° = full extension). The fatiguing tasks at the two knee joint angles were administered in randomized order.

### 2.3 MVC

Isometric knee extension MVC was measured on the right leg using a custom‐designed dynamometer (M‐12297‐3; Takei Scientific Instruments Co. Ltd., Niigata, Japan) mounted on a force transducer (LTZ‐100KA; Kyowa Electronic Instruments Co. Ltd., Tokyo, Japan), as previously reported [19]. During the knee extension tasks, the pelvis was secured to the seat with a strap at a hip joint angle of 110° (upright position = 180°), and the ankle was attached to a pad connected to the force transducer. The right knee joint was maintained at either 90° (flexed) or 140° (extended). The participants kept their arms folded across their chests. After at least a 2 min rest following submaximal contractions, the MVC test was performed three times. The MVC test consisted of three phases: a rising phase (2–3 s), a sustained phase (≥ 3 s), and a relaxation phase [19]. The participants were encouraged to exert maximal effort by the supervisors. Knee extension force was recorded at 400 Hz using an analog‐to‐digital converter (PowerLab 16SP; ADInstruments, Melbourne, Australia) and stored on a personal computer (Mac Mini; Apple Inc., Cupertino, CA, US). MVC force was identified as the peak value for each contraction. If the two highest forces differed by more than 5%, an additional trial was performed. The exerted force was multiplied by each participant's lower leg length to convert it to torque.

### 2.4 Fatiguing Task

Following a 10 min rest period, the participants performed intermittent isometric knee extensions consisting of a 5 s contraction and a 5 s relaxation in the same posture used during the MVC tests. Exercise began at 30% of MVC and increased by 10% every five contractions up to 70% of MVC, continuing until task failure [16]. Task failure was defined as the point at which, for three consecutive exertions, the produced force fell below the target force. Throughout the exercise, both the produced and target force were displayed on a personal computer monitor to provide visual feedback. The participants timed their contractions and relaxations in response to auditory cues from a metronome.

### 2.5 Intramuscular Blood Flow

Intramuscular blood flow was measured using power Doppler ultrasonography (LOGIQ e Premium; GE Healthcare, Wauwatosa, WI, USA) equipped with a 12‐MHz linear‐array probe (probe width, 3.8 cm), as previously described [15, 16, 17]. The probe was placed at the midpoint between the lateral epicondyle and the greater trochanter of the right thigh. It was positioned perpendicular to the estimated longitudinal axis of the vastus lateralis using a custom‐made styrene frame, and transverse images were acquired. The probe was covered with ample transducer gel to ensure acoustic contact without deforming the skin surface. For power Doppler measurements, all ultrasonography settings were kept constant, with acquisition parameters as follows: frequency, 6.3 MHz; gain, 30.5 dB; and depth, 7 cm [15, 16, 17]. The entire imaging field was displayed within the Doppler color area. Power Doppler images shown on the ultrasonography monitor were stored on a personal computer (ENVY; Hewlett‐Packard Japan, Tokyo, Japan) via a capture device (DVI2USB 3.0; Epiphan Video, Ottawa, ON, Canada) in AVI format at 10 frames/s.

**FIGURE 1:**
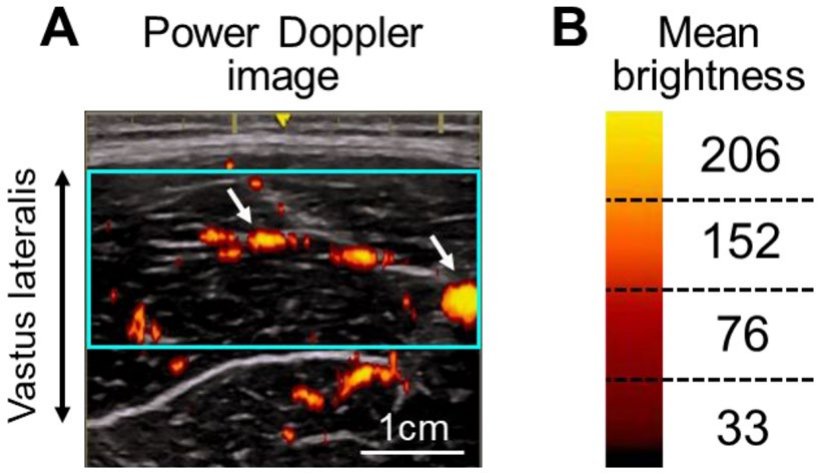
(A) Representative power Doppler image at exhaustion in flexion and (B) brightness division. The arrows indicate the distribution of intramuscular blood flow. The outlined area represents the region of interest.

Intramuscular blood flow was analyzed using custom‐designed software (S‐22028 version 1.0.3; Takei Scientific Instruments Co. Ltd.) developed to calculate the number of pixels representing the power Doppler signal within the image [15, 16, 17]. The analysis followed three steps:

1. Selection of region of interest (Figure 1A)

The region of interest in the vastus lateralis was selected using rectangular sections taken from a frame without muscle contraction or visible movement artifacts. As much muscle as possible was included while avoiding obvious fascia.

2 Calculation of mean brightness value (Figure 1B)

The color bar was divided into four segments, and the mean brightness for each level was calculated.

3 Evaluation of intramuscular blood flow

The mean brightness values were multiplied by the relative area of the signal at each level. Intramuscular blood flow was then expressed as the total across all four levels.

### 2.6 Muscle Deoxygenation

We assessed muscle deoxygenation in the vastus lateralis using a near‐infrared spectroscopy (NIRS) system (Hb14; Astem Co. Ltd., Kanagawa, Japan) equipped with a dual‐wavelength (770 nm and 830 nm) light‐emitting diode. The NIRS probe consisted of one emitter and two detectors, with optode distances of 2 cm and 3 cm. Oxyhemoglobin + myoglobin, deoxyhemoglobin + myoglobin (deoxy‐[Hb + Mb]), and total hemoglobin + myoglobin (total‐[Hb+Mb]) were determined from light attenuation at 770 nm and 830 nm and analyzed using algorithms based on a modified Beer‐Lambert law [20]. Subcutaneous fat thickness under the optode was measured using B‐mode ultrasonography (LOGIQ e Premium; GE Healthcare). The average subcutaneous fat thickness of the subjects was 0.42 ± 0.14 cm. By adjusting the scattering coefficient, subcutaneous fat thickness (i.e., path length) was used to minimize measurement error and to determine the relative change in Hb + Mb and the absolute StO_2_ by NIRS on a personal computer (ENVY; Hewlett‐Packard Japan) [21]. The NIRS system provided absolute StO_2_ values using relative absorption coefficients derived from the slope of light attenuation over a distance calculated from two focal points. The NIRS probe was positioned distal to the ultrasound probe used for intramuscular blood flow measurement, aligned perpendicular to the estimated longitudinal axis of the vastus lateralis. It was secured with elastic therapeutic tape and double‐sided adhesive tape to prevent interference from ambient light. NIRS signals were sampled at 2 Hz and transferred to a personal computer (ENVY; Hewlett‐Packard Japan) via Bluetooth 2.0.

### 2.7 mV̇O_2_

mV̇O_2_ was estimated using the following equation: mV̇O_2_ = intramuscular blood flow × O_2_ extraction fraction. Intramuscular blood flow was determined by power Doppler ultrasonography (LOGIQ e Premium; GE Healthcare). The O_2_ extraction fraction was estimated from the ratio of deoxy‐[Hb + Mb] relative to total‐[Hb + Mb] derived from NIRS (Hb14; Astem Co. Ltd.) [22].

### 2.8 Electrocardiogram

Electrocardiogram recordings were obtained using a three‐lead electrocardiograph (BSM‐3400; Nihon Kohden, Tokyo, Japan) and stored on a personal computer (Mac Mini; Apple Inc.) at 400 Hz via an analog‐to‐digital converter (PowerLab 16SP; ADInstruments). Heart rate (HR) was computed from each R–R interval. HR was measured as an index of exercise load.

### 2.9 Data Analysis

The resting values for all parameters were averaged over 3 min, excluding the first and last minute of the 5 min baseline period before exercise. Intramuscular blood flow and NIRS data were averaged during the relaxation phases, which were identified by reviewing the power Doppler video frame by frame at each contraction, and expressed as absolute changes (Δ) from baseline. HR was averaged over 5 s during the relaxation phases to avoid contraction‐induced artifacts. Exhaustion values were averaged over the final three contractions in which participants could no longer maintain the target force.

### 2.10 Statistical Analysis

All values are presented in tables and figures as mean ± standard deviation. Student's paired *t*‐test was used to compare MVC torque, and the Wilcoxon signed‐rank test was used to compare endurance time between knee joint angles. A two‐way (knee joint angle × exercise intensity) analysis of variance with repeated measures was conducted to examine differences in the parameters. When the analysis of variance indicated a significant effect, Bonferroni's post hoc test was applied. Statistical analyses were performed using IBM SPSS Version 27.0 (IBM Corp., Tokyo, Japan). The significance level was set at *p* < 0.05.

## 3 Results

### 3.1 MVC Torque, Endurance Time, and HR

**FIGURE 2:**
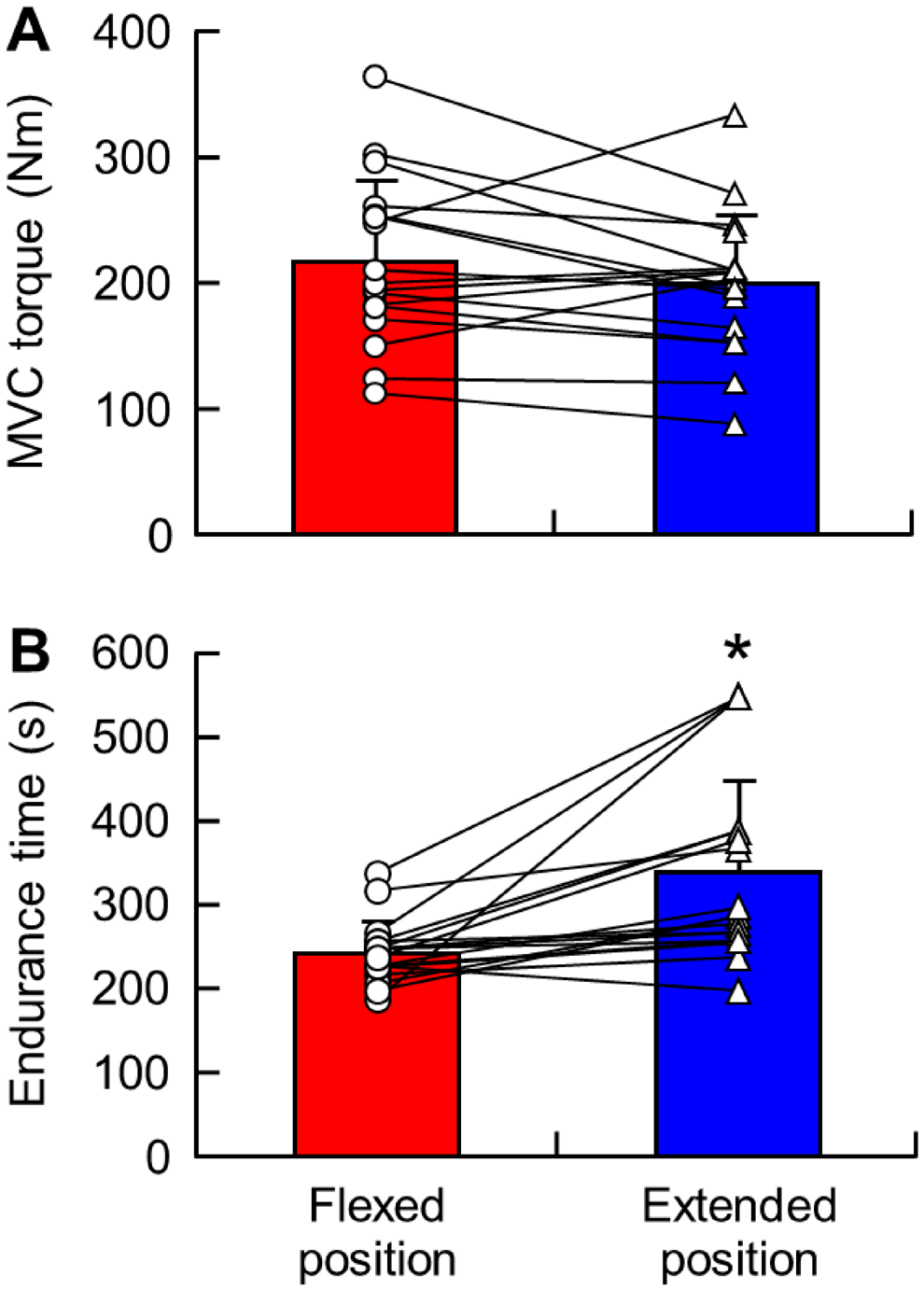
(A) MVC torque and (B) endurance time in the flexed and extended positions. *p < 0.05 vs. flexed position. MVC, maximal voluntary contraction.

There were no significant differences in MVC torque between knee joint angles (*p* = 0.139) (Figure 2A). However, endurance time was significantly longer in the extended than in the flexed position (*p* = 0.002) (Figure 2B). Changes in HR during the fatiguing tasks are shown in Table 1. A technical error resulted in the loss of HR data for one participant in the flexion trial. There were significant knee joint angle‐by‐exercise intensity interactions (*F* = 2.33, *p* = 0.043) and a main effect of exercise intensity (*F* = 30.58, *p* < 0.0001). Compared with baseline, HR increased significantly from 50% MVC to exhaustion in the flexed position (*p* = 0.033) and at 70% MVC and exhaustion in the extended position (*p* = 0.039). No significant differences in HR between knee joint angles were observed (*p* = 0.235).

**TABLE 1:** Heart rate during exercise.

	Baseline	Exercise (%MVC)					Exhaustion
		30	40	50	60	70	
Flexed position	74.3 ± 12.1	80.7 ± 12.7	83.5 ± 11.9	89.0 ± 12.8*	94.9 ± 14.5*	101.9 ± 18.8*	104.2 ± 16.9*
Extended position	74.9 ± 12.0	79.0 ± 9.6	80.2 ± 9.6	84.1 ± 11.3	90.2 ± 12.5	96.6 ± 13.9*	101.5 ± 14.8*

*Note:* Values are means ± SD. Unit = beats/min. Flexed position; *n* = 16, Extended position; *n* = 17. MVC, maximal voluntary contraction.

* *p* < 0.05, vs. baseline.

### 3.2 Absolute Changes in Intramuscular Blood Flow and NIRS Data

**FIGURE 3:**
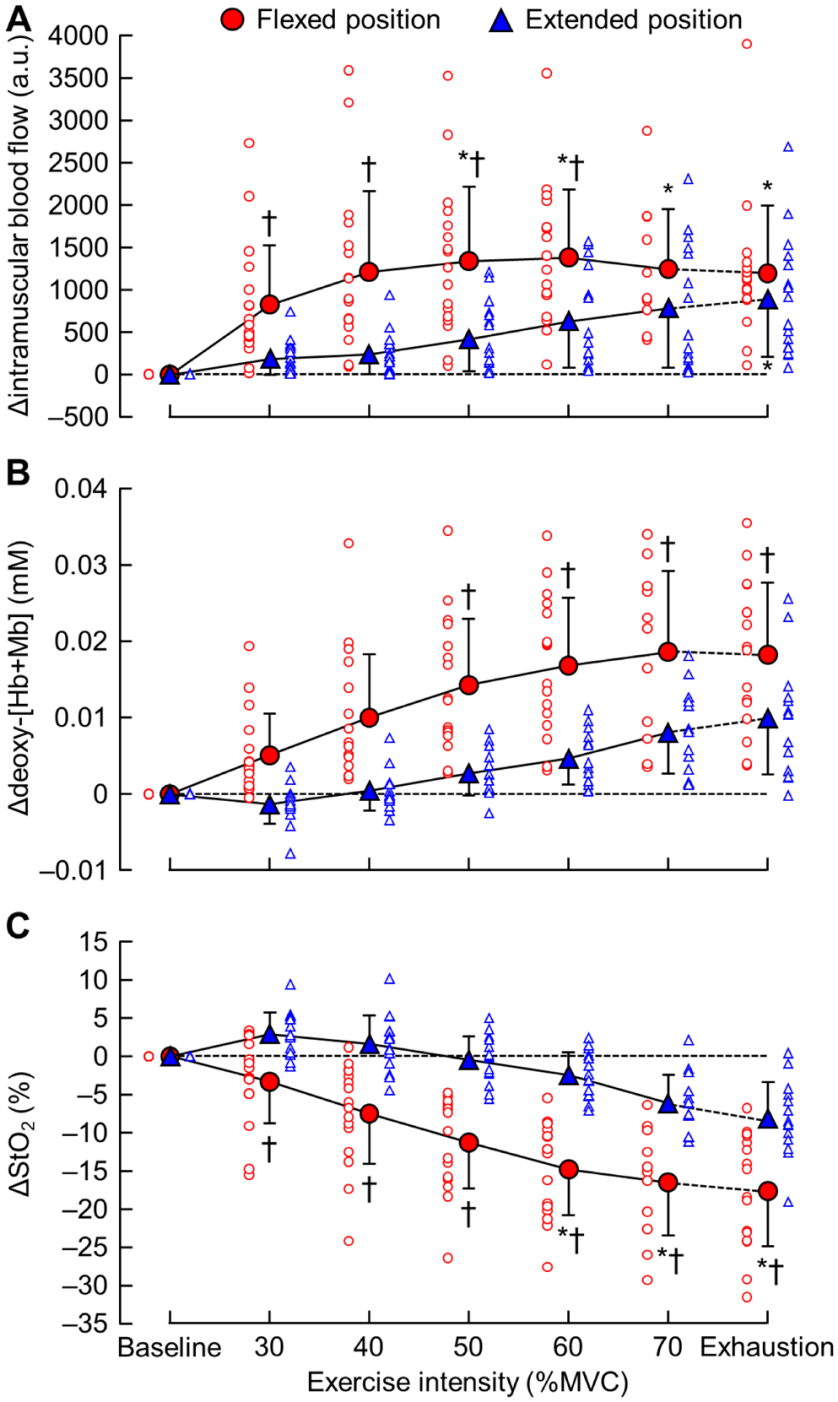
Absolute changes from baseline (Δ) for (A) intramuscular blood flow, (B) deoxy‐[Hb + Mb], and (C) StO_2_ in the flexed and extended positions. *p < 0.05 vs. baseline. †*p* < 0.05 vs. extended position. a.u., arbitrary unit; deoxy‐[Hb + Mb], deoxyhemoglobin + myoglobin; StO_2_, muscle oxygen saturation; MVC, maximal voluntary contraction.

Figure 3 shows the absolute changes from baseline (Δ) in intramuscular blood flow, deoxy‐[Hb + Mb], and StO_2_. NIRS data were missing for one participant in the flexed condition and three in the extended condition because of technical issues. Significant knee joint angle‐by‐exercise intensity interactions (*F* = 4.20, *p* = 0.001) and main effects of both knee joint angle (*F* = 17.49, *p* = 0.002) and exercise intensity (*F* = 12.90, *p* < 0.0001) were found for Δintramuscular blood flow. Compared with baseline, Δintramuscular blood flow increased significantly from 50% MVC to exhaustion in the flexed position (*p* = 0.027) and only at exhaustion in the extended position (*p* = 0.044). When comparing knee joint angles, Δintramuscular blood flow was significantly higher in the flexed than in the extended position from 30% to 60% MVC (*p* = 0.006).

Significant knee joint angle‐by‐exercise intensity interactions (*F* = 4.61, *p* = 0.001) and main effects of knee joint angle (*F* = 9.69, *p* = 0.017) and exercise intensity (*F* = 17.92, *p* < 0.0001) were also observed for Δdeoxy‐[Hb + Mb]. Compared with baseline, Δdeoxy‐[Hb + Mb] did not significantly change in either the flexed (*p* = 0.063) or extended position (*p* = 0.183). However, Δdeoxy‐[Hb + Mb] was significantly greater in the flexed than in the extended position from 50% MVC to exhaustion (*p* = 0.022).

For ΔStO_2_, significant knee joint angle‐by‐exercise intensity interactions (*F* = 6.47, *p* < 0.0001) and main effects of knee joint angle (*F* = 16.09, *p* = 0.005) and exercise intensity (*F* = 31.63, *p* < 0.0001) were observed. ΔStO_2_ decreased significantly compared with baseline at 60% MVC and above in the flexed position (*p* = 0.014). In comparisons between knee joint angles, ΔStO_2_ was significantly lower in the flexed than in the extended position above 30% MVC (*p* = 0.026).

### 3.3 Changes in mV̇O_2_

**FIGURE 4:**
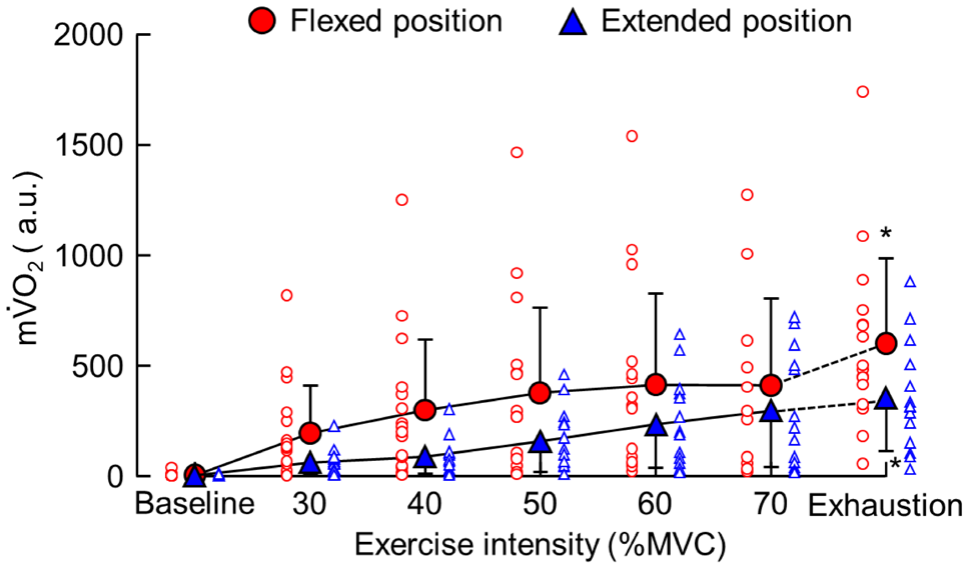
Changes in mV̇O_2_ in the flexed and extended positions. *p < 0.05 vs. baseline. mV̇O_2_, muscle oxygen consumption; a.u., arbitrary unit; MVC, maximal voluntary contraction.

The changes in mV̇O_2_ are shown in Figure 4. There were no significant knee joint angle‐by‐exercise intensity interactions (*F* = 1.47, *p* = 0.212) and main effect of knee joint angle (*F* = 0.21, *p* = 0.661). However, the main effect of exercise intensity (*F* = 22.48, *p* < 0.0001) was observed. mV̇O_2_ in the flexed and extended position was significantly higher than baseline at exhaustion (*p* = 0.015, *p* = 0.21, respectively). mV̇O_2_ did not significantly differ between knee joint angles (*p* = 0.144).

## 4 Discussion

We compared intramuscular blood flow and muscle deoxygenation between the flexed (90°) and extended (140°) knee positions during intermittent fatiguing isometric knee extension. The primary findings were that in the flexed compared with the extended position, Δintramuscular blood flow was significantly greater from 30% to 60% MVC, and ΔStO_2_ was significantly lower at all exercise intensities. These results indicate that knee joint angle influences changes in intramuscular blood flow and muscle deoxygenation at submaximal intensities during intermittent isometric knee extensions.

In this study, we analyzed intramuscular blood flow using a novel method in which intramuscular blood flow was evaluated as the sum of the products of mean brightness and relative area (Figure 1). Previous studies assessed intramuscular blood flow based on the relative area within the region of interest calculated from binarized power Doppler images [15, 16, 17, 23]. However, this binarization method cannot reflect the brightness of the Doppler signal, which varies depending on blood volume [18]. The approach used here provides a more detailed dataset of intramuscular blood flow derived from power Doppler ultrasonography.

MVC torque during isometric knee extension varies depending on knee joint angle, with the highest torque typically observed between 100° and 130° [24, 25, 26]. In the present study, no significant differences in MVC were found between joint angles (Figure 2A), likely because the tested angles were outside this optimal range. Prior studies have also reported differences in fatigue development at specific knee joint angles, [6, 7] and endurance time was significantly longer in the extended than in the flexed position here as well (Figure 2B).

The incremental intermittent protocol used in the present study was used in our previous work [16]. Although the methods for analyzing intramuscular blood flow differed between our previous study and this study, the results showed that the increase in intramuscular blood flow at knee flexion reached a submaximal plateau [16]. There is concern that measurements at high intensities may be influenced by preceding exercise. Our previous study examined the relationship between exercise intensity and intramuscular blood flow using a randomized intensity order design, resulting in a submaximal plateau [15]. This suggests that the present findings are unlikely to be influenced by preceding exercise during intermittent incremental isometric contractions.

The main findings of this study–that intramuscular blood flow was significantly higher at submaximal intensities and StO_2_ was significantly lower at all intensities in the flexed position (Figure 3)–suggest that microcirculation varies depending on mechanical and/or structural factors. To our knowledge, differences in intramuscular blood flow responses according to joint angle have not been examined previously. Muscle deoxygenation, however, has been shown to be more pronounced at a flexed angle, [27] consistent with our findings. Increases in vastus lateralis activity are associated with increases in intramuscular blood flow, with coefficients of determination ranging from 0.71 to 0.96 [28, 29]. We did not measure vastus lateralis activity here, but prior studies have not found differences in muscle activity between the flexed (90°) and extended (140°–150°) positions [2, 3, 4, 5, 6, 7]. Therefore, it is unlikely that muscle activation accounts for the differences in intramuscular blood flow and StO_2_.

No significant difference in mV̇O_2_ between joint angles was observed (Figure 4), which contradicts some previous studies reporting higher mV̇O_2_ in the flexed position [2, 3, 4, 5]. Although these previous studies assessed mV̇O_2_ by the slope of deoxy‐[Hb + Mb] during arterial occlusion, we calculated mV̇O_2_ from the product of intramuscular blood flow and deoxy‐[Hb + Mb]/total‐[Hb + Mb]. Deoxy‐[Hb + Mb]/total‐[Hb + Mb] is an indicator of deoxygenation that accounts for local blood volume [22]. The differences in mV̇O_2_ between the present and previous study may be attributable to variations in the methods for calculating mV̇O_2_. Although MVC torque did not significantly differ between knee joint angles (Figure 2B), the internal knee moment arm varies with knee joint angles. The internal knee moment arm has been shown to be greater in the extended than in the flexed position [30]. Thus, actual contractile force at the muscle level may have been higher in the flexed than in the extended angle despite similar external torque output. In addition, internal muscle force increases with muscle length, independent of the exerted torque level [31]. Kubo et al. [32] reported that internal vastus lateralis force at 80° was 2.3 times greater than at 130°. Therefore, increased contractile force and internal muscle force may be greater in the flexed position, and these mechanical factors may have restricted intramuscular blood flow, potentially requiring increased intramuscular blood flow to achieve the equivalent mV̇O_2_.

The sarcomere length of the vastus lateralis is longer at the flexed than at the extended knee joint angle [8, 9]. Changes in capillary structure and microcirculatory dynamics influenced by sarcomere length may help explain differences in intramuscular blood flow and StO_2_ between joint angles. Capillaries envelop muscle fibers; when fibers are stretched, most capillaries stretch similarly [33]. Poole et al. [12] demonstrated that capillary luminal diameter decreases and red blood cell velocity slows in the lengthened rat spinotrapezius muscle. They also showed that the proportion of capillaries with sustained perfusion declined from 95.9% at the shortest sarcomere length to 56.5% when sarcomeres were lengthened [12]. Muscle stretching reduces StO_2_ in humans, suggesting that stretching‐induced alterations in skeletal muscle microvasculature restrict intramuscular blood flow [34]. The response of intramuscular blood flow and StO_2_ during the relaxation phase reflects oxygen demand during the muscle contraction phase [35]. Thus, intramuscular blood flow may have been higher and StO_2_ lower during the relaxation phases in the present study because microvascular perfusion tended to be more restricted in the flexed position during the muscle contraction phase, where the vastus lateralis was more lengthened than in the extended position. In addition, capillary tortuosity depends on sarcomere length. Because microvascular vessels run not strictly parallel but somewhat tortuously along the muscle fiber axis, capillary tortuosity increases as sarcomeres shorten [36]. Tortuous vessels can promote blood accumulation within the microvasculature and enhance oxygen exchange by reducing diffusion distance between capillaries and muscle cells. Structural variations in the microvasculature depending on knee joint angle may therefore influence skeletal muscle microcirculation, including intramuscular blood flow and muscle deoxygenation.

By contrast, the lack of significant differences in intramuscular blood flow between the flexed and extended positions at 70% MVC and exhaustion may reflect the slow rise in intramuscular blood flow during the relaxation phase. Our previous study reported that intramuscular blood flow required more than 10 s to reach its peak after a 5 s isometric knee extension at 70% MVC, [15] suggesting that peak intramuscular blood flow may not have been reached within the 5 s relaxation phases at intensities above 70% MVC in the present study.

Intramuscular blood flow showed high variability (Figure 3), as observed in a previous study using power Doppler ultrasonography [37]. This inter‐individual variability observed in intramuscular blood flow responses may be attributable to physiological factors. Individual differences in muscle architecture (e.g., fascicle length and pennation angle), vascular structure, and physical fitness levels could influence the degree of reactive hyperemia during the relaxation phase [38, 39]. However, these remain speculative, necessitating further investigation into factors related to variability.

This study has several limitations. First, we measured parameters only in the vastus lateralis, and we did not assess neuromuscular activation in the quadriceps femoris. Neuromuscular activation of the quadriceps varies with knee joint angle and contraction modality [7, 40, 41]. For example, although normalized median frequency in the vastus lateralis and vastus intermedius at the flexed position decreases with repeated maximal knee extensions, the values for the vastus lateralis, vastus intermedius, and vastus medialis remain stable in the extended position throughout the exhaustive task [7]. The dynamics of intramuscular blood flow and muscle deoxygenation may therefore differ if other quadriceps muscles or other contraction modalities (e.g., isokinetic contraction) are examined. Another limitation is that the measurement regions for power Doppler ultrasonography and NIRS were not strictly identical. Muscle deoxygenation was assessed in superficial regions, whereas intramuscular blood flow was evaluated at a depth of approximately 3 cm in the vastus lateralis.

In conclusion, we non‐invasively examined the effect of knee joint angle on intramuscular blood flow and muscle deoxygenation during fatiguing isometric knee extension. This approach demonstrated that intramuscular blood flow was significantly higher and StO_2_ was significantly lower in the flexed than in the extended knee joint angle, despite equivalent force production between angles. These findings indicate that knee joint angle influences changes in intramuscular blood flow and muscle deoxygenation during intermittent isometric knee extensions, suggesting that metabolic requirements differ according to mechanical and/or structural factors.

### 4.1 Perspective

We provide novel findings showing that intramuscular blood flow and muscle deoxygenation are affected by knee joint angle even when equivalent force output levels are produced. By combining power Doppler ultrasonography and NIRS, we evaluated oxygen supply and consumption non‐invasively and continuously in a single muscle under different joint angles. These results suggest that metabolic demands differ according to mechanical and/or structural factors. These findings provide practical insight into optimizing training and rehabilitation programs. Specifically, selecting a more flexed knee position may be advantageous when the goal is to amplify metabolic stress or microvascular stimulation in the vastus lateralis during intermittent isometric knee extension. By contrast, a more extended knee position may be preferable when the goal is to limit fatigue while maintaining the same relative torque, such as during early‐phase strength training or rehabilitation. Understanding these joint angle–dependent microvascular responses may help practitioners optimize the balance between training stimulus and fatigue according to individual performance goals or clinical needs.

## Author Contributions

Conception of design of the work: K.I., K.K., N.T. and H.A. Acquisition, analysis, or interpretation of the data for the work: K.I., K.K., Y.K., N.T. and H.A. Drafting of the work or revising it critically for important intellectual content: K.I., K.K., Y.K., N.T. and H.A. All authors approved the final version of the manuscript and agree to be accountable for all aspects of the work in ensuring that questions related to the accuracy or integrity of any part of the work are appropriately investigated and resolved. All persons designated as authors qualify for authorship, and all those who qualify for authorship are listed.

## Funding

Grant‐in‐Aid for Challenging Research Exploratory from the Ministry of Education, Culture, Sports, Science and Technology Grants (22 K19728) and JST SPRING grant (JPMJSP2125).

## Conflicts of Interest

The authors declare no conflicts of interest.

## Data Availability

The data that support the findings of this study are available on request from the corresponding author. The data are not publicly available due to privacy or ethical restrictions.
